# Orthobunyavirus Antibodies in Humans, Yucatan Peninsula, Mexico

**DOI:** 10.3201/eid1810.120492

**Published:** 2012-10

**Authors:** Bradley J. Blitvich, Rungrat Saiyasombat, Lourdes G. Talavera-Aguilar, Julian E. Garcia-Rejon, Jose A. Farfan-Ale, Carlos Machain-Williams, Maria A. Loroño-Pino

**Affiliations:** Iowa State University College of Veterinary Medicine, Ames, Iowa, USA (B.J. Blitvich, R Saiyasombat);; and Universidad Autónoma de Yucatán Centro de Investigaciones Regionales Dr. Hideyo Noguchi, Mérida, México (L.G. Talavera-Aguilar, J.E. Garcia-Rejon, J.A. Farfan-Ale, C. Machain-Williams, M.A. Loroño-Pino)

**Keywords:** orthobunyavirus, Cache Valley virus, Cholul virus, South River virus, serology, surveillance, Mexico, Yucatan Peninsula, viruses, *Suggested citation for this article*: Blitvich BJ, Saiyasombat R, Talavera-Aguilar LG, Garcia-Rejon JE, Farfan-Ale JA, Machain-Williams C, et al. Orthobunyavirus antibodies in humans, Yucatan Peninsula, Mexico. Emerg Infect Dis [Internet]. 2012 Oct [*date cited*]. http://dx.doi.org/10.3201/eid1810.120492

## Abstract

We performed a serologic investigation to determine whether orthobunyaviruses commonly infect humans in the Yucatan Peninsula of Mexico. Orthobunyavirus-specific antibodies were detected by plaque reduction neutralization test in 146 (18%) of 823 persons tested. Further studies are needed to determine health risks for humans from this potentially deadly group of viruses.

We previously reported the isolation of Cache Valley virus (CVV), Kairi virus (KRIV), Cholul virus (CHLV), and South River virus (SOURV) from mosquitoes in the Yucatan Peninsula of Mexico (*1*–*3*). Antibodies to CVV, CHLV, and SOURV were also detected in livestock in this region (*4*). These viruses belong to the genus *Orthobunyavirus* (*5*). All viruses in this genus possess a tripartite, single-stranded, negative-sense RNA genome.

CVV is a recognized human pathogen (*5*) that has been linked to severe encephalitis and multiorgan failure. KRIV has not been implicated as a cause of human disease, although antibodies to this virus have been detected in humans in Argentina (*6*). Recent data suggest that CHLV is a reassortant that acquired its small RNA segment from CVV and medium and large RNA segments from Potosi virus (POTV) (*1*). No clear evidence exists for human susceptibility to infection with CHLV or SOURV. However, diagnostic laboratories rarely test for orthobunyavirus infection; therefore, the true disease incidence and seroprevalence of these viruses remains to be determined. Because orthobunyaviruses comprise a neglected but potentially deadly group of viruses and recent studies have provided evidence of orthobunyavirus activity in the Yucatan Peninsula (*1*–*4*), we investigated whether orthobunyaviruses commonly infect humans in this region.

## The Study

Serum samples were obtained from 823 febrile patients at the Secretaria de Salud de Yucatán and other health institutions in Merida during January–October 2007. The patients resided in all 3 states of the Yucatan Peninsula of Mexico: Yucatan (n = 809), Quintana Roo (n = 8) and Campeche (n = 6). The study was approved by the Institutional Biosafety Committees at Iowa State University (Ames, IA, USA) and the Universidad Autónoma de Yucatán (Mérida, Mexico).

All serum samples were examined at a dilution of 1:20 by plaque reduction neutralization test (PRNT) by using CVV (strain CVV-478), and PRNTs were performed as described (*7*). A subset of serum samples with antibodies that neutralized CVV were titrated and further analyzed by PRNT by using CVV, CHLV (strain CHLV-Mex07), KRIV (strain KRIV-Mex07), SOURV (strain NJO-94f), Maguari virus (strain BeAr7272), and Wyeomyia virus (strain prototype). All of these viruses belong to the Bunyamwera (BUN) serogroup except SOURV, which belongs to the California (CAL) serogroup.

Titers were expressed as the reciprocal of highest serum dilutions yielding >90% reduction in the number of plaques (PRNT_90_). For etiologic diagnosis, the PRNT_90_ antibody titer for each virus was required to be >4-fold greater than that to the other viruses tested.

Antibodies that neutralized CVV were detected in 146 (18%) of 823 study participants. The mean ages of patients with and without antibodies that neutralized CVV were 32.0 and 22.3 years, respectively. Logistic regression analysis showed that the risk for infection increased significantly with age (p = 0.0001).

Serum samples from 50 seropositive patients were titrated and analyzed by comparative PRNT to identify the orthobunyaviruses responsible for these infections. Six persons were seropositive for CVV, 5 for CHLV, and 1 for SOURV or a SOURV-like virus; 38 had antibodies to an undetermined orthobunyavirus (Table). Because SOURV was the only CAL serogroup virus used in this study, and another CAL serogroup virus may have been responsible for the infection, the person who had a SOURV PRNT_90_ titer >4-fold than that to the other viruses tested received a conservative PRNT diagnosis of seropositive for SOURV or a SOURV-like virus. Because interserogroup crossreactivity of neutralizing antibodies to viruses in the BUN and CAL serogroups has not been seen, the 17 persons with antibodies that neutralized SOURV and >1 of the BUN serogroup viruses might have been exposed to >1 viruses from each serogroup.

**Table:** Endpoint titers of serum samples collected from persons in Mexico and analyzed by using comparative PRNT*

Patient ID no.	Demographic characteristics			PRNT_90_ titers						Diagnosis
	Residence	Age, y/sex		CVV	CHLV	KRIV	SOURV	MAGV	WYOV	
28	Yucatan	18/M		80	20	–	–	40	–	UND
34	Yucatan	39/F		80	20	–	–	20	–	CVV
52	Quintana Roo	39/F		160	–	–	40	80	–	UND
54	Yucatan	32/M		40	20	–	–	40	–	UND
62	Yucatan	44/M		40	–	–	20	20	–	UND
72	Yucatan	23/M		80	–	–	–	40	–	UND
81	Yucatan	60/F		20	20	–	20	–	–	UND
92	Yucatan	13/M		160	20	–	20	80	–	UND
93	Yucatan	42/F		40	20	–	–	20	–	UND
113	Yucatan	24/F		20	–	–	–	–	–	UND
114	Yucatan	29/F		40	–	–	–	20	–	UND
120	Yucatan	60/F		20	–	–	320	–	–	SOURV or SOURV-like virus
159	Yucatan	54/M		20	–	–	–	20	–	UND
161	Yucatan	53/M		20	20	–	–	–	–	UND
163	Yucatan	27/F		160	80	40	–	40	–	UND
167	Yucatan	16/M		160	–	–	–	40	–	CVV
183	Yucatan	69/F		20	–	–	–	20	–	UND
184	Yucatan	34/M		160	40	–	–	80	–	UND
185	Yucatan	25/F		20	–	–	–	–	–	UND
192	Campeche	54/F		80	40	–	–	20	–	UND
193	Yucatan	16/F		80	–	–	–	20	–	CVV
194	Yucatan	69/F		20	–	–	20	–	–	UND
200	Yucatan	3/F		40	–	–	40	40	–	UND
205	Yucatan	53/M		40	–	–	20	20	–	UND
208	Yucatan	57/F		20	160	–	–	–	–	CHLV
210	Yucatan	42/M		20	–	–	–	20	–	UND
224	Yucatan	34/M		20	20	–	20	–	–	UND
234	Yucatan	39/F		20	80	–	–	20	–	CHLV
236	Yucatan	74/F		20	–	–	20	–	–	UND
386	Yucatan	14/F		20	–	–	20	–	–	UND
388	Yucatan	60/M		160	1,280	160	–	40	–	CHLV
389	Yucatan	5/M		20	–	–	20	–	–	UND
390	Yucatan	33/M		40	20	40	20	40	–	UND
392	Yucatan	22/M		20	–	–	–	–	–	UND
393	Yucatan	29/M		40	20	–	–	40	–	UND
396	Yucatan	34/F		80	–	–	–	20	–	CVV
397	Yucatan	32/M		80	–	–	–	40	–	UND
399	Yucatan	27/M		320	1,280	160	–	320	–	CHLV
400	Yucatan	37/F		20	–	–	–	–	–	UND
401	Yucatan	30/F		160	–	–	–	40	–	CVV
402	Yucatan	18/M		20	–	–	–	20	–	UND
403	Yucatan	50/F		20	20	–	–	–	–	UND
407	Yucatan	27/M		80	20	–	–	40	–	UND
408	Yucatan	40/F		20	–	–	–	–	–	UND
412	Yucatan	60/M		20	320	80	40	40	–	CHLV
415	Yucatan	17/F		20	–	–	20	–	–	UND
420	Yucatan	16/F		160	–	–	–	20	–	CVV
429	Yucatan	37/F		20	–	–	–	–	–	UND
442	Yucatan	10/F		20	40	–	20	–	–	UND
455	Yucatan	30/F		20	–	–	20	20	–	UND

*PRNT, plaque reduction neutralization test; CVV, Cache Valley virus; CHLV, Cholul virus; KRIV, Kairi virus; SOURV, South River virus; MAGV, Maguari virus, WYOV, Wyeomyia virus; –, titer <20; UND, undetermined orthobunyavirus.

CHLV and POTV share the same medium RNA segment, so antibodies for these viruses cannot be differentiated by PRNT. Furthermore, antibodies to CHLV and POTV cannot be differentiated by complement fixation test (*4*). Thus, we cannot dismiss POTV as a possible cause of infection in some or all of the study participants who were seropositive for CHLV. However, it appears more likely that these persons had been infected with CHLV because this virus has been isolated in the Yucatan Peninsula, whereas no direct evidence has been found for POTV in this region.

As already noted, serum samples from 38 (76%) of the study participants analyzed by comparative PRNT had antibodies to an undetermined orthobunyavirus. Most of these persons had low PRNT_90_ titers; the highest PRNT_90_ titer for 29 of these persons did not exceed 40. Because neutralizing antibody levels decline over time, these findings may indicate that many of these infections occurred years ago, and the trace amounts of neutralizing antibodies that remained were insufficient to yield a >4-fold difference between the titers of the virus responsible for the infection and the other viruses used in the PRNTs. Another explanation is that some of these persons had been infected with an orthobunyavirus not included in the PRNTs, although all orthobunyaviruses known to occur in the Yucatan Peninsula were represented.

## Conclusions

We found 18% of the 823 Yucatan residents participating in our study had evidence of orthobunyavirus exposure. This number is presumably an underestimate; additional seropositive persons might have been identified if the initial PRNTs had not been restricted to CVV. In particular, additional seropositive persons likely would have been detected if SOURV was used in the initial PRNTs, because a screening algorithm that includes only a BUN serogroup virus would likely miss many CAL serogroup virus infections. Nevertheless, we provide evidence that orthobunyaviruses commonly infect humans in the Yucatan Peninsula.

Previous serosurveys have provided information on the seroprevalence of orthobunyaviruses in humans in the United States. For example, antibodies that neutralized CVV were detected in 42/356 (12%) residents in Maryland and Virginia in 1961–1963 (*8*). Antibodies that neutralized Maguari virus or Tensaw virus were detected in 71/≈300 humans in Florida in the 1980s (*9*); as observed in our study, the highest PRNT titers for most of the seropositive persons in that study did not exceed 40.

All persons in our study cohort initially sought care for unspecified fever; however, we could not determine whether any of these febrile illnesses were a direct consequence of orthobunyavirus infection. The detection of acute orthobunyavirus infections is limited because no IgM-capture ELISA for orthobunyavirus diagnosis exists. PRNTs can be used to identify recent orthobunyavirus infections when paired acute and convalescent serum samples are available, but for our study, only single serum samples were available from each participant. Orthobunyavirus viremias in humans are transient and of low magnitude, which makes reverse transcription PCR ineffective for the detection of orthobunyavirus RNA in serum samples. However, a duplex reverse transcription PCR was recently developed for the detection of CVV RNA in human cerebrospinal fluid (*10*).

In conclusion, we provide evidence that orthobunyaviruses commonly infect humans in the Yucatan Peninsula. These viruses are also a common cause of infection of livestock in this region (*4*). Our findings underscore the need to determine whether orthobunyaviruses represent an unrecognized cause of illness in humans and vertebrate animals in Mexico.
